# Isolation and identification of a distinct strain of Culex Flavivirus from mosquitoes collected in Mainland China

**DOI:** 10.1186/1743-422X-9-73

**Published:** 2012-03-27

**Authors:** Wang Huanyu, Wang Haiyan, Fu Shihong, Liu Guifang, Liu Hong, Gao Xiaoyan, Song Lizhi, Simon Rayner, Xu Aiqiang, Liang Guodong

**Affiliations:** 1 State Key Laboratory for Infectious Disease Prevention and Control, Department of Viral Encephalitis, Institute for Viral Disease Control and Prevention, Chinese Center for Disease Control and Prevention, Beijing 102206, People's Republic of China; 2 Institute for Immunization Program, Shandong province Center for Disease Control and Prevention, Jinan, Shandong 250014, People's Republic of China; 3 Bioinformatics Group, State Key Laboratory for Virology, Wuhan Institute of Virology, Chinese Academy of Sciences, Wuhan, Hubei 430071, People's Republic of China

**Keywords:** CulexFlavivirus, Genotype, Mosquitoes

## Abstract

**Background:**

Culexflavivirus (CxFV) is an insect specific virus that has been isolated from *Culexpipiens, Culexquinquefasciatus, Culextritaeniorhynchus *and other *Culex *mosquitoes. It is a novel flavivirus isolated in Asia, North America, Central America and Africa. Phylogenetic analysis indicates that, based on the envelope gene (E gene) sequence, the worldwide CxFV strains can be divided into two genotypes.

**Result:**

A virus (SDDM06-11) was isolated from *Culexpipiens *collected in Shandong Province, China in 2006. The strain caused cytopathic effect (CPE) in *Aedesalbopictus *(C6/36) cells by 3 days post-infection and immunofluorescence assay (IFA) showed a reaction with Japanese encephalitis virus (JEV) polyclonal antibodies. Phylogenetic analysis of the E gene sequence showed CxFV formed two genotypes with the SDDM06-11 strain assigned to genotype 1. Analysis of the E gene nucleotide homology showed the virus possessed characteristic amino acids at specific sites. The nucleotide homology of the open reading frame (ORF) was 94.8%-95.1% between SDDM06-11 and isolates from Japan, Iowa and Texas, and 90.2%-90.5% between SDDM06-11 and isolates from Uganda and Mexico.

**Conclusion:**

In this paper we report the first isolation and identification of an isolate of CxFV in mainland China. Phylogenetic analysis indicates the isolate belongs to genotype 1. Our findings provide insight into the occurrence of CxFV in *Culex *mosquito populations and its distribution on a global scale.

## Background

The genus *Flavivirus *in the family *Flaviviridae *is a group of single stranded positive sense RNA viruses that are primarily arthropod borne [1]. Phylogenetic analysis classifies these viruses into mosquito-borne, tick-borne, no known vector and viruses that are specific to insects, but which do not infect vertebrates [2,3]. This final group includes cell fusing agent virus (CFAV) [4], Kamiti River virus (KRV)[5] and Culexflavivirus (CxFV)[6].

CxFV is an insect-specific virus that can replicate in C6/36 cells but not in other mammalian cell lines. CxFV was first isolated in 2003 in Japan (in species *Culexpipiens *and *Culextritaeniorhynchus*) and then in Indonesia in 2004 (*Culexquinquefasciatus*)[6]. Subsequently the virus has been isolated in North America: Iowa and Texas in the United States [7,8] (2007-2008, *Culexquinquefasciatus, Culexrestuans*); Central America: Guatemala (2006, *Culexquinquefasciatus*) [9], the Yucatan peninsula in Mexico [10,11] (2007-2008, *Culexquinquefasciatus, Culexinterrogator*); Trinidad in the West Indies [8] (2008, *Culexquinquefasciatus*); and Uganda, Africa [12] (2008, *Culexquinquefasciatus*). E gene phylogenetic analysis of these isolates showed that they can be clearly divided into two genotypes [8,9].

Relatively few flaviviruses have been isolated in China, the most common of which are Japanese encephalitis virus (JEV), dengue virus (DENV) and tick-borne encephalitis virus (TBEV) with presence of associated disease symptoms [13,14]. In recent years, a novel insect-specific Flavivirus was isolated from *Aedesvexans *in rural corrals in Chaoyang city in Liaoning province in China [15], but the distribution and significance of these insect specific viruses remains unclear in China.

In this study, we report the isolation and identification of a distinct strain of CxFV from *Culexpipiens *mosquitoes collected in Shandong Province in China in 2006 and perform nucleotide sequencing analysis in order to compare with other CxFV isolates previously collected from around the world.

## Methods

### Mosquito collection

Mosquitoes used in this study were collected in August in 2006 in Dongming county, Shandong province, China. Mosquitoes were collected using light traps (Hubei Lucky Star Environment Protection Co., Ltd.) in fields, pig farms and dwellings. The traps were placed before sunset and mosquitoes were collected from the traps the following morning after sunrise. Collected mosquitoes were frozen for 30 min at -20°C then placed on an ice plate to determine mosquito species and exclude blood-fed and/or male mosquitoes [16]. The mosquitoes were sorted according to species with 50 to 100 adults per pool, transported on dry ice and then stored in liquid nitrogen in the laboratory.

### Cell culture and virus isolation

Mosquito cell line C6/36 and baby hamster kidney (BHK-21) cell line were used for virus isolation. The two cell lines were cultured with Eagle's minimum essential medium (MEM) (Sigma-Aldrich, USA) with 10% heat-inactivated fetal bovine serum (FBS) (Sigma), 2% non-essential amino acid (Sigma), 100 U/ml penicillin (Gibco, Invitrogen, USA), 100 μg/ml streptomycin (Gibco), and maintained at 28°C (for mosquito cells) and 37°C (for mammalian cells) with 5% CO_2_.

Pools of mosquitoes were homogenized using a Mixer Mill (Tissuelyser, Qiagen, Germany) and 3 mm stainless steel beads in 2 mL sterile plastic tubes and 1.5 mL of MEM containing 2% fetal bovine serum, 2 mM L-glutamine, 50 units/mL Penicillin and 100 ug/mL Streptomycin (Gibco Cat No 15140). Samples were centrifuged at 12,000 rpm for 20 minutes at 4°C. Then, 200 μl of the clarified homogenates were inoculated into single 5.5 cm^2 ^Nunc tubes (Nunclon, Denmark) spread with a monolayer of C6/36 and BHK-21 cells respectively for 1 h at constant temperature. After discarding and refreshing with 500 μl medium, the cell cultures were incubated with the same conditions for 6-7 days. Cytopathic effects (CPE) were checked every 8 hours after incubation for 24 hours and observation over the next 6-7 days. At 70% CPE (3 days after post-infection), the culture supernatants were harvested and cellular debris was removed by centrifugation at 12,000 rpm. The supernatants were stored at -80°C until identification.

### Indirect immunofluorescence assay

Virus isolate in cell culture was identified by indirect immunofluorescence assay (IFA). Infected and uninfected cell suspensions were applied to Teflon-coated 10-well slides, which were then air dried and fixed in acetone. The IFA was conducted with a panel of antiserum with fluorescein isothiocyanate-conjugated goat anti-mouse IgG as the second antibody (Sigma) by previously described procedures [17]. The panel of polyclonal antibody (JEV-GSS) against JEV was prepared by our laboratory [18].

### RT-PCR and nucleotide sequencing

Viral RNA was extracted from 140 μl of virus culture stocks using the QIAamp viral RNA extraction kit (Qiagen, Valencia, CA, USA) in accordance with the manufacturer's protocol and stored at -70°C until use. Briefly, purified RNA was used as template to finish the reverse transcription-polymerase chain reactions (RT-PCR) for cDNA synthesis using Ready-To-Go™ You-Prime First-Strand Beads (Amersham Biosciences, Piscatway, NJ, USA) and supplied random hexanucleotide primers. *Flavivirus *genus-specific primer sets [3] were used for identification. Then ten overlapping primers (Table 1) were designed from the Tokyo strain genome sequence (GenBank number: AB262759) to amplify the complete open reading frame (ORF) nucleotide sequence of the viral genomic RNA.

**Table 1:** Degenerate primer sequences used for viral screening and primer sets used for SDDM06-11

Primer	**Position **^**a**^	Sequence, 5'-3'	Polarity
CxFV-SD-1-F	21-39	TGGTTACACCGCAGATTG	Sense

CxFV-SD-1-R	1198-1217	GATTGTAGGGCTGGGTTGAG	Reverse

CxFV-SD-2-F	1034-1049	AACGGACTTCTTGAGTTTCGC	Sense

CxFV-SD-2-R	2197-2217	GCCTTGGTGTAGACAAAGTATC	Reverse

CxFV-SD-3-F	2002-2020	GCAAGGTTGGAGAATGGC	Sense

CxFV-SD-3-R	3238-3256	GACCTTGAAGTGAAATACCC	Reverse

CxFV-SD-4-F	3034-3055	GTCTAAAGCAAACCACATTCC	Sense

CxFV-SD-4-R	4233-4255	CGGTCCGTAAGTTCCTTCTAAT	Reverse

CxFV-SD-5-F	3880-3989	CTATGCTGTGACGCATCCTG	Sense

CxFV-SD-5-R	6232-6254	AAGCCACAAATAGTCCATACAG	Reverse

CxFV-SD-6-F	6048-6069	ACTCAGTGCTGTTTCAAGG	Sense

CxFV-SD-6-R	7204-7226	CTCGTCCTGTTATCTCATCGTC	Reverse

CxFV-SD-7-F	7021-7039	GATTTCCTGGGAGACGAT	Sense

CxFV-SD-7-R	8319-8337	TCTCGCAACCTCTTCACG	Reverse

CxFV-SD-8-F	8012-8033	TTCTGTTGTAAGGTGCTGTCT	Sense

CxFV-SD-8-R	9326-9344	GGTTCTTCCCATTCGTCA	Reverse

CxFV-SD-9-F	9140-9159	CTGGACCAAGTGACTGACC	Sense

CxFV-SD-9-R	10332-10350	TGGCTGCGAGGGTGACTT	Reverse

CxFV-SD-10-F	10016-10038	AGTTTGATTGGTGAGCGGGACA	Sense

CxFV-SD-10-R	10794-10815	CCCGCAACAAGTCTCCTAACG	Reverse

^a ^The nucleotide position was according to the CxFV strain Tokyo genome.

To avoid any potential contamination, JEV RNA and water were used as positive and negative controls respectively at all stages. RT-PCR products were purified using a commercial kit (ExoSAP-IT PCR Purification Kit, USB, USA). PCR products were sequenced using a PRIMS Ready Reaction Dyedeoxy Terminator Cycle Sequencing Kit on an ABI Prism 3100 Avant Genetic Analyser (Applied Biosystems, California, USA) using the PCR primer sets listed in Table 1. Dye terminator sequencing was used for both strands with double coverage and the results were inspected for accuracy and quality. After sequencing we got the ATGC software package (GENETYX Corp., Tokyo, Japan) was used to complete the sequence assembly.

Sequence data for the E and ORF gene of strain SDDM06-11 were deposited in GenBank with accession number JF938690 and JQ518484, respectively.

### Sequence analysis and phylogenetic comparisons

Additional CxFV sequences were downloaded from GenBank, a list of the 49 CxFV isolates used in the analyses with origin and year of isolation is shown in Table 2. Nucleotide and amino acid sequence alignments were generated by CLUSTAL_X version 1.8 [19]. The optimal nucleotide substitution model was estimated as the General Time Reversible with rate heterogeneity among sites and invariable sites using Akaike Information Criterion as implemented in jModeltest v.0.1.1 (http://darwin.uvigo.es/software/jmodeltest.html). The phylogenetic tree was computed with the GTR + I + G model using the maximum likelihood approach with the PHYML software package [20]. The statistical significance of the phylogeny was estimated by the nonparametric bootstrap analysis with 100 replicates.

**Table 2:** Details of Culexflavivirus (CxFV) strains used in this study

**No**.	Strain	Year	Geographic Location			**GenBank accession no**.
					
			Country	Location	Source/Host	
1	SDDM06-11*	2006	China	Dongming	*Culexpipiens*	JF938690

2	Tokyo	2003	Japan	Tokyo-Shinjuku	*Culexpipiens*	NC_008604/AB262759

3	NIID-21-2	2004	Japan	Tokyo-Shinjuku	*Culexpipiens*	AB377213

4	Narita	2003	Japan	Chiba-Narita	*Culexpipiens*	AB262760

5	Toyama	2003	Japan	Toyama	*Culexpipiens*	AB262761

6	Hokkaido	2003	Japan	Hokkaido-Abashiri	*Culexpipiens*	AB262762

7	Osaka	2003	Japan	Osaka	*Culexpipiens*	AB262763

8	Mie-Cp	2004	Japan	Mie-Tsu	*Culexpipiens*	AB262764

9	Morioka	2003	Japan	Iwate-Morioka	*Culexpipiens*	AB262765

10	Mie-Ct	2004	Japan	Mie-Tsu	*Culextritaeniorhynchus*	AB262767

11	Surabaya	2004	Indonesia	Surabaya	*Culexquinquefasciatus*	AB262766

12	Izabal	2006	Guatemala	Puerto Rico Izabal	*Culexquinquefasciatus*	EU805805

13	HOU24518	2008	USA	Texas-Harris county	*Culexquinquefasciatus*	FJ502995

14	HOU24519	2008	USA	Texas-Harris county	*Culexrestuans*	FJ502996

15	HOU24284	2008	USA	Texas-Harris county	*Culexrestuans*	FJ502997

16	HOU24471	2008	USA	Texas-Harris county	*Culexrestuans*	FJ502998

17	HOU24516	2008	USA	Texas-Harris county	*Culexquinquefasciatus*	FJ502999

18	HOU24559	2008	USA	Texas-Harris county	*Culexquinquefasciatus*	FJ503001

19	TR3115	2008	West Indies, Trinidad	Champs Fleur	*Culexquinquefasciatus*	FJ503002

20	HOU24522	2008	USA	Texas-Harris county	*Culexquinquefasciatus*	FJ503000

21	TR3116	2008	West Indies, Trinidad	Champs Fleur	*Culexquinquefasciatus*	FJ503003

22	CxFV-Mex07	2007	Mexico	Yucatan State	*Culexquinquefasciatus*	EU879060

23	1064	2007	USA	Iowa	*Culexpipiens*	FJ663026

24	380	2007	USA	Iowa	*Culexpipiens*	FJ663027

25	383	2007	USA	Iowa	*Culexpipiens*	FJ663028

26	635	2007	USA	Iowa	*Culexpipiens*	FJ663029

27	318	2007	USA	Iowa	*Culexpipiens*	FJ663030

28	377	2007	USA	Iowa	*Culexpipiens*	FJ663031

29	599	2007	USA	Iowa	*Culexpipiens*	FJ663032

30	657	2007	USA	Iowa	*Culexpipiens*	FJ663033

31	Iowa07	2007	USA	Iowa	*Culexpipiens*	FJ663034

32	Uganda08	2008	Uganda	Entebbe	*Culexquinquefasciatus*	GQ165808

33	T955	2008	Mexico	Yucatan Peninsula	*Culexinterrogator*	GU289683

34	M2168	2008	Mexico	Yucatan Peninsula	*Culexquinquefasciatus*	GU289684

35	M2313	2008	Mexico	Yucatan Peninsula	*Culexquinquefasciatus*	GU289685

36	M2605	2008	Mexico	Yucatan Peninsula	*Culexquinquefasciatus*	GU289686

37	M2614	2008	Mexico	Yucatan Peninsula	*Culexquinquefasciatus*	GU289687

38	M2617	2008	Mexico	Yucatan Peninsula	*Culexquinquefasciatus*	GU289688

39	M2618	2008	Mexico	Yucatan Peninsula	*Culexquinquefasciatus*	GU289689

40	M2630	2008	Mexico	Yucatan Peninsula	*Culexquinquefasciatus*	GU289690

41	M2635	2008	Mexico	Yucatan Peninsula	*Culexquinquefasciatus*	GU289691

42	M2636	2008	Mexico	Yucatan Peninsula	*Culexquinquefasciatus*	GU289692

43	M2637	2008	Mexico	Yucatan Peninsula	*Culexquinquefasciatus*	GU289693

44	M2644	2008	Mexico	Yucatan Peninsula	*Culexquinquefasciatus*	GU289694

45	M2645	2008	Mexico	Yucatan Peninsula	*Culexquinquefasciatus*	GU289695

46	M2648	2008	Mexico	Yucatan Peninsula	*Culexquinquefasciatus*	GU289696

47	M2650	2008	Mexico	Yucatan Peninsula	*Culexquinquefasciatus*	GU289697

48	M2656	2008	Mexico	Yucatan Peninsula	*Culexquinquefasciatus*	GU289698

49	M2663	2008	Mexico	Yucatan Peninsula	*Culexquinquefasciatus*	GU289699

50	M2665	2008	Mexico	Yucatan Peninsula	*Culexquinquefasciatus*	GU289700

*Sequence of SDDM06-11 was used in the present study.

Neighbor Joining (NJ) and Maximum Likelihood (ML) trees were also estimated for the nucleotide alignments using PHYLIP, v3.6-alpha (http://atgc.lirmm.fr/phyml) and MEGA v5.1 with empirical base frequencies, and a gamma distribution to estimate rate variation among sites. Bootstrap values were determined for 100 replicates.

The trees were rooted using a CRFV (GenBank accession no. NC001564) sequence as the outgroup. Analysis of nucleotide and translated amino acid sequence identities for the E and ORF sequences was performed using the GeneDoc and Lasergene software packages (DNASTAR Inc, USA).

## Results

### Virus isolation and identification

**Figure 1:**
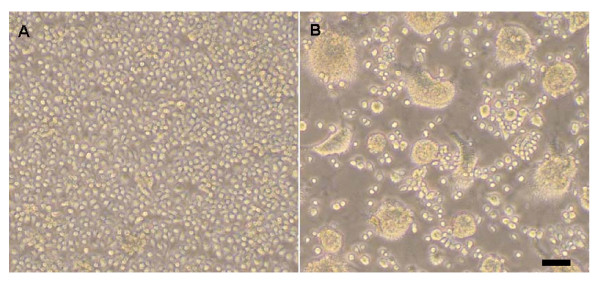
**Phase contrast photomicrographs of control and infected C6/36 cells after infection**. (**A**) Control cells. (**B**) Cells 3 days after infection with SDDM06-11. Note the extensive cell fusion and syncytia formation in the infected cells. Microscope settings Ocular: 10; Lens: 10X. Scale bar, 50 μm.

**Figure 2:**
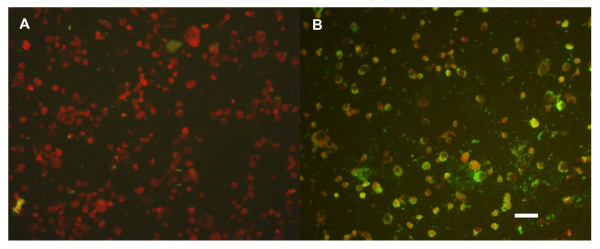
**Immunofluorescence assay (IFA) of SDDM06-11 using JEV polyclonal antibody (JEV-GSS)**. (**A**) C6/36 cell control (uninfected) stained by IFA, using JEV polyclonal antibody (JEV-GSS). (**B**) SDDM06-11 antigen in infected C6/36 cells, as detected by IFA, using JEV polyclonal antibody (JEV-GSS). Scale bar, 50 μm.

A total of 80 pools consisting of 4118 *Culextritaeniorhynchus*, 839 *Culexpipiens *and 27 *Anopheles sinensis *were collected from Dongming county, Shandong province, China, from August 9 to 11, 2006 and were processed for virus isolation. A virus isolate was identified that caused CPE in C6/36 cells and which was characterized by marked syncytia, aggregation and fusion by day 3 post-infection (Figure 1A and 1B); no CPE was observed in BHK-21 cells. Antigen IFA using JEV polyclonal antibody (JEV-GSS) identified the isolate as a flavivirus (Figure 2A and 2B). RT-PCR amplification using flavivirus primers FU1 and cFD2 [3]. BLASTN analysis against the nr database at NCBI showed the highest scoring hits were all from CxFV with 95% similarity and an E-value of 0. The isolate was designated SDDM06-11.

### Analysis of the E gene sequence

Table 3 shows a summary of the nucleotide and amino acid identities for the E gene according to genotype and geographical location amongst strains used in the phylogenetic analysis.

**Table 3:** Percentage nucleotide/amino acid identity for the envelope genes among Culexflavivirus isolates from different countries and genotypes

				GENOTYPE 1				GENOTYPE 2		
		**China**	**Japan**	**Indonesia**	**Texas**	**Iowa**	**Mexico**	**Guatemala**	**Trinidad**	**Uganda**

	**China**	--	**94.6**	**94.8**	**94.5**	**94.4**	**89.4**	**89.7**	**89.6**	**90.2**
	
	**Japan**	98.6	--	**97.5**	**97.4**	**97.4**	**89.1**	**89.3**	**89.3**	**90.2**
	
**GEN 1**	**Indonesia**	98.4	99.3	--	**97.4**	**97.3**	**89.9**	**90.1**	**90.1**	**90.9**
	
	**Texas**	98.6	99.5	99.3	--	**99.6**	**89.8**	**89.9**	**89.5**	**90.6**
	
	**Iowa**	98.6	99.5	99.3	100	--	**90**	**90.2**	**89.8**	**90.8**

	**Mexico**	97.9	97.4	97.2	97.4	97.4	--	**99.2**	**98.4**	**97.9**
	
	**Guatemala**	97.9	97.4	97.2	97.4	97.4	100	--	**98.4**	**97.9**
	
**GEN 2**	**Trinidad**	97.9	97.4	97.2	97.4	97.4	100	100	--	**98**
	
	**Uganda**	97.9	97.4	97.2	97.4	97.4	99.5	99.5	99.5	--

Percentage nucleotide and amino acid homology between and amongst genotypes and countries; nucleotide divergence is displayed in bold type in upper triangle, amino acid homology is displayed in regular type in lower triangle. Alignments were performed using the following CxFV isolates: SDDM06-11 (China), NIID-21-2 (Japan), Surabaya (Indonesia), TX 24518 (Texas), 380 (Iowa) belonging to Genotype 1 and CxFV-Mex07 (Mexico), Izabal (Guatemala), TR3116 (Trinidad) and Uganda08 (Uganda) belonging to Genotype 2.

Specifically, the nucleotide identity between SDDM06-11 and genotype 1 strains (Japan, Indonesia and North America) (Iowa and Texas) was 94.4%-94.8%, and the corresponding identity of SDDM06-11 with genotype 2 (Uganda, Trinidad, Guatemala and Mexico) was 89.4%-90.2%. The identity between genotype 1 and genotype 2 was 89.1%-90.9%. The within genotype identity for genotype 1 was 94.4%-99.6% and within genotype 2 was 97.9%-99.2%. The E gene amino acid identity amongst all the CxFV strains was 97.2%-100%(Table 3).

Examination of the amino acid sequence for the E gene indicated that each genotype contained site-specific mutations (Table 4). A total of ten mutations were identified that were specific to genotype I and 2; these occurred at E-60 (Val), E-64 (Phe), E-117 (Gly), E-185 (His), E-236 (Val), E-326 (Arg), E-340 (Ile), E381 (His) E-407E (Leu), E-423 (Ile). Mutations that were unique to SDDM06-11 (i.e., that were not present in genotype 1 or 2) occurred at E-125 (Val) and E-414 (Val).

**Table 4:** Deduced amino acid substitutions for CxFV by genotype in the E gene

**Genotype**^**1**^						**Residue**^**2**^						
	
	E-60	E-64	E-117	E-125	E-185	E-236	E-326	E-340	E-381	E-407	E-414	E-423
G1	V (Val)	F (Phe)	G (Gly)	I (Ile)	H (His)	V (Val)	R (Arg)	**V (Val)**	H (His)	**F (Phe)**	F (Phe)	**V (Val)**

G2	**A**^**3 **^**(Ala)**	**Y (Tyr)**	**E (Glu)**	I (Ile)	**Y (Tyr)**	**I (Ile)**	**K (Lys)**	I (Ile)	**Y (Tyr)**	L (Leu)	F (Phe)	I (Ile)

SDDM06-11	V (Val)	F (Phe)	G (Gly)	**V (Val)**	H (His)	V (Val)	R (Arg)	I (Ile)	H (His)	L (Leu)	**V (Val)**	I (Ile)

^1^G1: Japan and Indonesia, USA:TX/Iowa; G2: Uganda, Guatemala, Mexico, West Indies;

^2 ^Amino acid positions are determined from the sequence alignment of the completely E sequenced isolates of CxFV.

^3 ^Boldface indicates deduced amino acid specific to that genotype.

### Analysis of the ORF gene sequence

The nucleotide and deduced amino acid sequences of the ORF of SDDM06-11 were compared with those of six other CxFV strains selected from the GenBank database. The nucleotide identity between SDDM06-11 and other genotype 1 sequences (Japan, Iowa and Texas) was 94.8%-95.1%, and between SDDM06-11 and genotype 2 sequences (Uganda and Mexico) was 90.2%-90.5%. The calculated amino acid identity for all the CxFV strains was 96.5%-98.2% (Table 5). The highest nucleotide identity occurred in the NS2a and NS2b genes, where the nucleotide identity between SDDM06-11 and genotype 1 strains was > 98% and > 96% respectively;. For genotype 2, the identity was > 94% and > 92% respectively. however, the amino acid homology was lower than ORF (Table 5).

**Table 5:** Nucleotide and amino acid of ORF sequence comparison (% sequence identity) of strain SDDM06-11 with selected CxFV isolates

SDDM06-11	C	PrM	E	NS1	NS2a	NS2b	NS3	NS4a	NS4b	NS5	Total
	
	417(139)^a^	429 (143)	1281 (427)	1107 (369)	690 (230)	381 (127)	1779 (593)	567 (189)	771 (257)	2667 (889)	10089 (3363)
**Tokyo**	94.5 (95.0)^b^	95.3 (98.6)	94.6 (98.6)	94.7 (99.2)	98.3 (97.8)	96.9 (94.5)	94.3 (97.5)	94.0 (98.4)	94.0 (97.3)	94.7 (98.7)	94.9 (98.0)

**NIID-21-2**	94.7 (95.0)	95.6 (99.3)	94.6 (98.6)	94.8 (99.2)	98.3 (97.8)	96.9 (94.5)	94.5 (97.6)	94.0 (98.4)	94.0 (97.3)	95.0 (99.1)	95.0 (98.2)

**Iowa07**	95.0 (94.2)	95.6 (99.3)	95.0 (98.8)	94.8 (99.2)	98.6 (98.3)	96.6 (94.5)	94.5 (97.5)	94.0 (98.4)	94.2 (97.3)	95.1 (99.3)	95.1 (98.2)

**HOU24518**	95.0 (95.0)	94.6 (98.6)	94.5 (98.6)	94.4 (99.2)	98.3 (98.3)	96.1 (95.3)	94.2 (97.5)	94.4 (99.5)	94.4 (96.9)	94.6 (98.7)	94.8 (98.1)

**CxFV-Mex07**	91.8 (89.9)	92.8 (98.6)	89.4 (97.9)	91.3 (98.1)	94.8 (93.9)	92.4 (93.7)	88.8 (96.5)	87.5 (96.3)	88.8 (95.7)	89.8 (97.5)	90.2 (96.6)

**UGANDA08**	92.3 (91.4)	93.0 (97.9)	90.2 (97.9)	92.0 (98.1)	94.8 (94.3)	92.9 (94.5)	89.3 (96.5)	88.4 (96.3)	87.7 (94.2)	89.8 (97.2)	90.5 (96.5)

^a ^Nucleotide number were ahead and amino acid number shown in brackets.

^b ^Percent nucleotide sequence homology were ahead and amino acid sequence homology shown in brackets.

### Phylogenetic analysis

**Figure 3:**
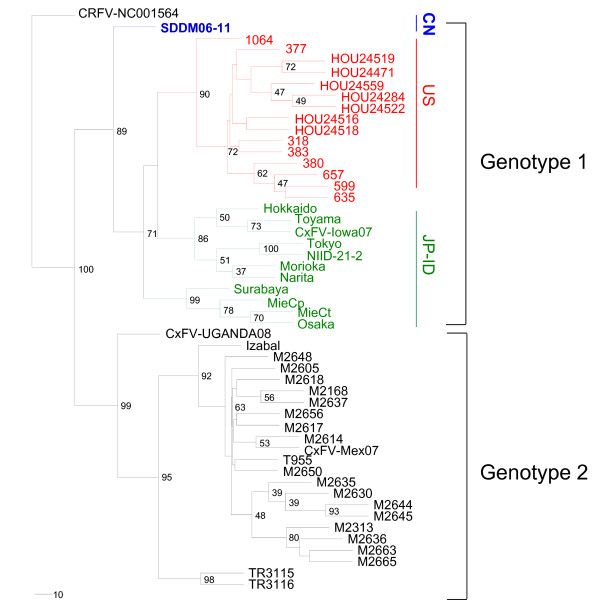
**Phylogenetic analysis based on the E gene of CxFV strain isolated in China**. Phylogenetic tree generated using the ML method. The tree was rooted by using CRFV (GenBank accession no. NC001564) sequence as the outgroup. Horizontal branch lengths are proportional to genetic distance; scale bars indicate a genetic distance of 10-nt substitutions per site. Isolate obtained in Shandong Province is shown in boldface and blue. Isolates from United States are shown in red and isolates from Japan and Indonesia are shown in green. See Table 2 for sequence information. CN: China; US: United States; JP: Japan; ID: Indonesia.

The estimated ML phylogenetic tree based on the E gene nucleotide sequence is shown in Figure 3 and is consistent with the trees estimated for the using the NJ and ML methods using MEGA v5.1 (data not shown). The tree shows the CxFV strains are divided into two distinct genotypes. Genotype 1 is divided into two clades, with clade 1 incorporating sequences from Japan and Indonesia and clade 2 containing samples from the United States (Texas and Iowa) [8]. Genotype 2 contains samples from Africa (Uganda), North America (Mexico), Central America (Guatemala) and the West Indies, (Trinidad) [7]. The newly isolated Chinese strain SDDM06-11 is placed within the genotype 1 clade; however, it appears to have branched from the remaining isolates at a relatively early time point, suggesting that it belongs to a distinct cluster (Figure 3).

## Discussion

We report here the first isolation of a strain of CxFV in China. This strain is distinct from other isolates collected from around the world, containing signature amino acid mutations and forming a separate cluster in the estimated phylogenetic tree. This sample appears therefore to represent a new cluster within genotype 1; this is also supported by the analysis of the amino acid substitutions in the E protein; many of the mutations were shared by these genotypes, whereas SDDM06-11 contains several unique substitutions.

Investigation of the CPE of this strain also produced distinctive results. Previous studies of CPE produced by CxFV in C6/36 cells found that some genotype 1 strains could generate marked CPE in cells within 6 to 7 days but no such changes have been observed for genotype 2 strains [7,9,10]. For the SDDM06-11 from China the CPE was similar to observations for genotype 1 [6,8], producing marked CPE but with a more rapid onset of effects (3-4 days) This difference may be a consequence of the detected amino acid changes in the E gene, but detailed experimental studies are necessary to gain further insight and understanding of the observed differences between the two genotypes.

The genotypes are formed with high bootstrap support and contain clear geographical subdivision, but on a global scale the geographical distribution is less clear with North America (Texas and Iowa), Japan and the Chinese isolate placed in genotype 1 and the Central America (Guatemala and Mexico), Trinidad and Africa strains placed in genotype 2. Similarly, the evolutionary relationship among the three genotypes is not apparent from the estimated phylogenetic tree. This is primarily because there are relatively few samples available for the analysis and only a partial evolutionary history of the virus can be recreated.

All CxFV samples to date have been isolated from members of the C*ulex *genus, with the majority of samples collected from *Culexpipiens *and *Culexquinquefasciatus*. Genotype 2 primarily consists of *Culexquinquefasciatus *isolates, whereas genotype 1 is primarily *Culexpipiens *and contains smaller numbers of other *Culex *species [6-11]. The SDDM06-11 belonging to genotype 1 is also isolated from *Culexpipiens*.

An earlier theoretical study of species diversification derived an estimate for the relationship between the distribution of internal nodes in a tree predicted from a limited sample set and the number of different viruses from which the sample set were selected [21]. When applied to the flaviviruses genus, it was estimated that are several thousand unsampled mosquito borne flaviviruses [21]. Thus further collection and evaluation of CxFV samples is necessary to gain further insight into the evolutionary history of the virus and the host range. It is likely that this will reveal greater complexity and structure (i.e. additional genotypes and new strains) in the CxFV phylogeny and host population.

## Conclusions

We have isolated and identified the CxFV strain in mainland China. Phylogenetic analysis places the isolate within genotype 1, but indicates it is quite distinct from other strains within this clade. This is further supported by analysis of the E gene and ORF sequence which shows the isolate has discrete difference in terms of nucleotide composition and amino acid mutations. Our findings suggest that CxFV may be widespread in *Culex *mosquito populations and could have a relevant role in the evolution of flaviviruses.

## Abbreviations

CPE: Cytopathic Effect; CxFV: CulexFlavivirus; IFA: immunofluorescence assay

## Competing interests

The authors declare that they have no competing interests.

## Authors' contributions

Wang Huanyu^1^, Wang Haiyan^2^, Fu Shihong ^1^, Liu Guifang^2^, Liu Hong^1^, Gao Xiaoyan^1^, Song Lizhi^2^, Simon Rayner^3^, Xu Aiqiang^2^, Liang Guodong^1^*

WHYu carried out mosquito collection, virus isolation, nucleic acid detection and sequencing, participated in the sequence alignment, phylogenetic analysis and drafted the manuscript. WHYan, LGF, SLZ and XAQ participated in the mosquito collection. FSH, LH and GXY participated in the virus isolation, nucleic acid detection and phylogenetic analysis. SR participated in discussions and data analysis. LGD conceived the study and participated in its design and coordination. All authors read and approved the final manuscript.

## Authors' information

Dr. Wang Huanyu, Ph.D., is an associate professor at the State Key Laboratory for Infectious Disease Prevention and Control, the Institute for Viral Disease Control and Prevention, Chinese Center for Disease Control and Prevention. His current research focuses on arbovirus and viral encephalitis, especially in Flavivirus.
